# Amoxicillin/clavulanic acid-induced pancreatitis: case report

**DOI:** 10.1186/s12876-018-0851-6

**Published:** 2018-08-02

**Authors:** Sana Chams, Skye El Sayegh, Mulham Hamdon, Sarwan Kumar, Vesna Tegeltija

**Affiliations:** Department of Internal Medicine, Wayne State University School of Medicine, Rochester Hills, MI USA

**Keywords:** Amoxicillin/clavulanic acid, Amoxicillin, Pancreatitis, Drug-induced pancreatitis

## Abstract

**Background:**

Acute pancreatitis is an acute inflammation of the pancreas that varies in severity from mild to life threatening usually requiring hospitalization. The true incidence of drug-induced pancreatitis (DIP) is indeterminate due to the inadequate documentation of case reports of DIP. Here we present the case of amoxicillin/clavulanic acid-induced pancreatitis in a previously healthy male after excluding all other causes of pancreatitis.

**Case presentation:**

A 58-year-old Caucasian man presenting for acute sharp abdominal pain with associated nausea and heaves. Pain was non-radiating and worsening with movement. Patient had no constitutional symptoms. The only medication he received prior to presentation was amoxicillin/clavulanic acid as prophylaxis for a dental procedure with his symptoms starting on day 9th of therapy. Laboratory studies revealed mild leukocytosis, increased levels of serum lipase, amylase, and C-reactive protein (CRP). Abdominal computed tomography (CT) was notable for acute pancreatitis with no pseudocyst formation. Hence, patient was diagnosed with mild acute pancreatitis that was treated with aggressive intravenous (IV) hydration and pain management with bowel rest of 2 days duration and significant improvement being noticed within 72 h. On further questioning, patient recalled that several years ago he had similar abdominal pain that developed after taking amoxicillin/clavulanic acid but did not seek medical attention at that time and the pain resolved within few days while abstaining from food intake. All other causes of pancreatitis were ruled out in this patient who is non-alcoholic, non-smoker, and never had gallstones. Abdominal ultrasound and magnetic resonance cholangiopancreatography (MRCP) eliminated out the possibility of gallstones, biliary ductal dilatation, or choledocholithiasis. Patient had no hypertriglyceridemia nor hypercalcemia, never had endoscopic retrograde cholangiopancreatography (ERCP), never took steroids, has no known malignancy, infection, trauma, or exposure to scorpions.

**Conclusion:**

This case describes a patient with DIP after the intake of amoxicillin/clavulanic acid and when all other common causes of acute pancreatitis were excluded. Only two other case reports were available through literature review regarding amoxicillin/clavulanic acid- induced pancreatitis.

We again stress on the importance of identifying and reporting cases of DIP to raise awareness among physicians and clinicians.

## Background

Acute pancreatitis is an acute inflammation of the pancreas that varies in severity from mild to life threatening usually requiring hospitalization. The predominant symptom is severe abdominal pain and diagnosis can be made through blood tests and imaging studies such as x-rays, ultrasound, and computed tomography (CT) scan. The major causes of acute pancreatitis are gallstones (30–60%) and heavy alcohol use (15–30%) in addition to other common causes: hypertriglyceridemia, hyperparathyroidism, endoscopic retrograde cholangiopancreatography (ERCP), trauma, pancreatic tumors, surgery, infections, and medications [1]. Of increasing interest is the drug-induced pancreatitis (DIP) which is less common (1–2%) even though true incidence is indeterminate due to the inadequate documentation of case reports of DIP where DIP is often undiagnosed, misdiagnosed, or underdiagnosed [1–4]. Here lies the importance of identifying cases of DIP in medical practice and the need for documenting and publishing such cases to increase the awareness among physicians regarding the side effects of most commonly used drugs and also to aid scientists and researchers in identifying the mechanism behind DIP. Here we present the case of amoxicillin/clavulanic acid-induced pancreatitis in a previously healthy male after excluding all other causes of pancreatitis.

We followed CARE reporting guidelines in publishing our case report.

## Case presentation

**Fig. 1:**
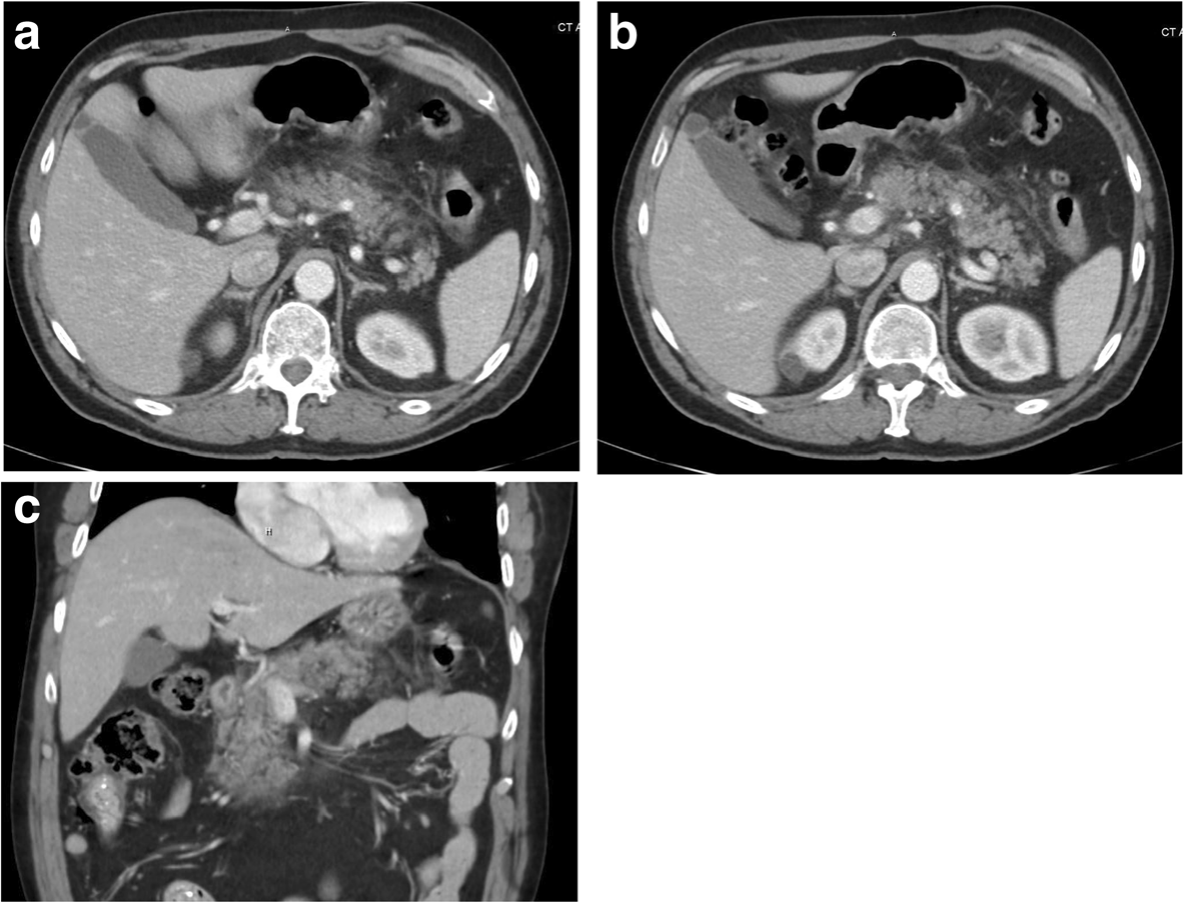
CT of the abdomen and pelvis. **a** and **b** Axial plane showing infiltration of the peripancreatic fat planes by soft tissue attenuation complicated with inflammation. No pancreatic ductal dilatation or discrete peripancreatic fluid collections observed. No stones in adjacent gall bladder. **c** Similar findings in coronal plane

A 58-year-old Caucasian man presented to the emergency department for acute abdominal pain. The abdominal pain was mainly in the epigastric area, was sharp in nature, with severity of 8/10, non-radiating, worsens with movement, and mildly improves with rest. The pain was associated with nausea and heaves. On review of systems he denied any constitutional symptoms (weight loss, fever, chills, weakness or fatigue), no cardiovascular, respiratory, neurological, musculoskeletal, hematological or endocrinological problems. Past medical history is only significant for hypothyroidism for which he takes levothyroxine. No previous surgeries done. Patient was not taking any medications except for Levothyroxine for hypothyroidism for the past 10 years. The only medication he received prior to presentation was amoxicillin/clavulanic acid as prophylaxis for a dental procedure (even though not indicated at that time) with dosage of 875 mg twice daily for a total of 10 days with his symptoms starting on day 9th of therapy and amoxicillin/clavulanic acid was discontinued on admission to hospital. On further questioning, patient recalled that several years ago he had similar abdominal pain that developed after taking amoxicillin/clavulanic acid but did not seek medical attention at that time and the pain resolved within few days while abstaining from food intake. He is a non-smoker, has never used recreational drugs, drinks only socially on certain occasions not exceeding twice a month and not exceeding 2 beers, 5% alcohol based, in one sitting (a total of 24 oz), and denies binge drinking. On admission, he was hemodynamically stable. His physical examination was noticeable for epigastric tenderness only. Laboratory studies revealed mild leukocytosis (white blood count (WBC): 13.5 × 10^9^/L), increased levels of serum lipase > 600 U/L, amylase: 1220 U/L, and CRP: 19.6 mg/dL. Abdominal CT was notable for acute pancreatitis with no pseudocyst formation (Fig. 1).

Based on clinical presentation and CT findings, patient was diagnosed with mild acute pancreatitis with Bedside Index of Severity in Acute Pancreatitis (BISAP) score of 0 (< 1% risk of mortality), which is characterized by the absence of organ failure and local or systemic complications. During his hospital stay, patient was managed with aggressive IV hydration and pain management with bowel rest of 2 days duration and significant improvement being noticed within 72 h after which patient was discharged home.

**Fig. 2:**
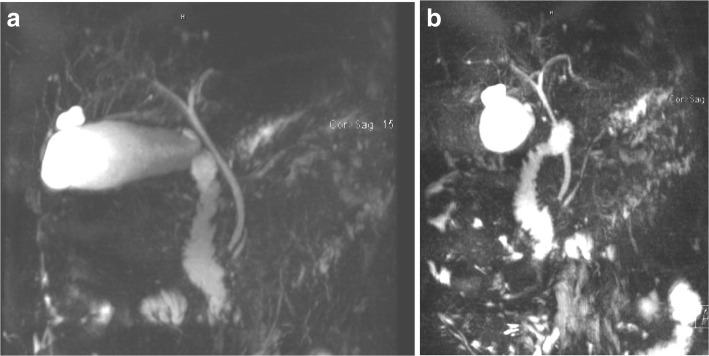
MRCP images. **a** Normal caliber CBD (common bile duct). **b** Normal caliber main pancreatic duct

**Fig. 3:**
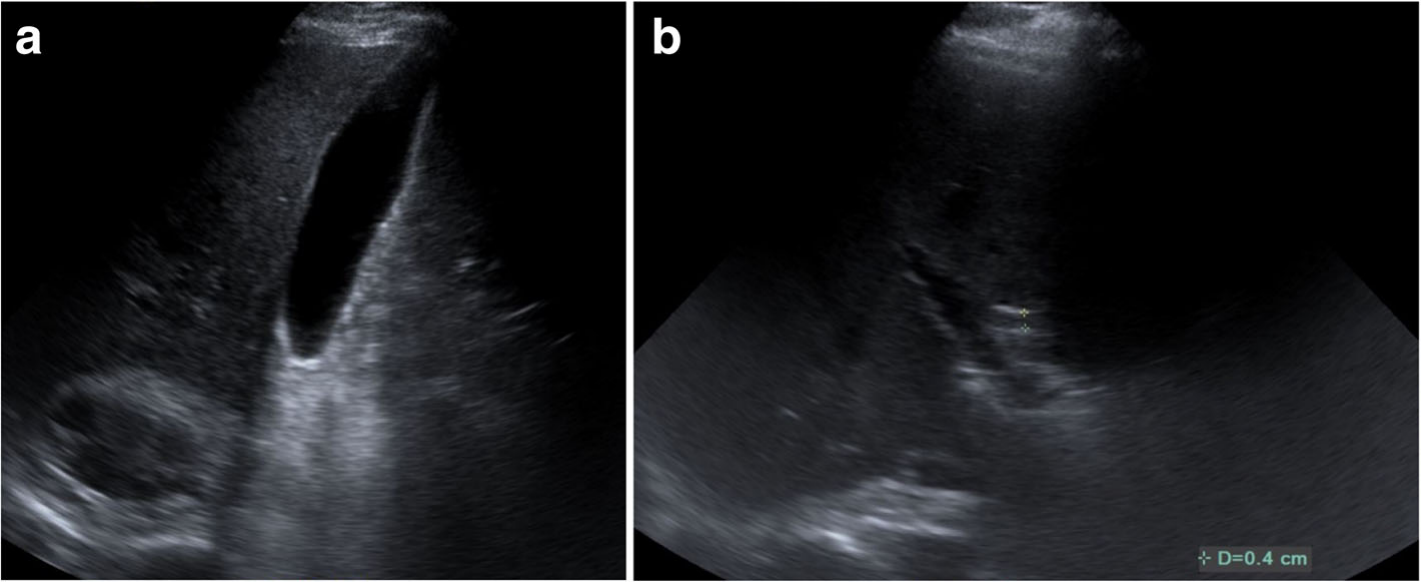
Abdominal ultrasound images. **a** Gallbladder without any stones. **b** Normal caliber common bile duct ≤ 7 mm demonstrated

In order to identify the cause of his acute pancreatitis, extensive history and workup was done with the help of the gastroenterology team to eliminate the most common causes of pancreatitis. Magnetic resonance cholangiopancreatography (MRCP) and abdominal ultrasound and eliminated out the possibility of gallstones, biliary sludge, biliary ductal dilatation, or choledocholithiasis (Figs. 2 and 3).

Endoscopic ultrasonography was done as outpatient by the gastroenterologist on the case and ruled out biliary microlithiasis. Patient had no hypertriglyceridemia (his triglyceride (TG): 142 mg/dL), never had endoscopic retrograde cholangiopancreatography (ERCP), no hypercalcemia (his corrected calcium (Ca): 9.3 mg/dL), no steroids taken, no known malignancy, no infection, no trauma, no exposure to scorpions. The most plausible link for his pancreatitis was his use of amoxicillin/clavulanic acid prior to presentation given that he had a similar presentation when he took the same antibiotic several years ago but was not diagnosed with pancreatitis since he did not seek medical attention at that time. Additionally, patient denied intake of any other penicillin agents. Table 1 summarizes our case’s timeline.

**Table 1:** Timeline Table

Relevant Past Medical History and Interventions		
Past medical history significant for hypothyroidism. Patient received amoxicillin/clavulanic acid as prophylaxis for a dental procedure (even though not indicated at that time) with dosage of 875 mg twice daily for a total of 10 days with his symptoms starting on day 9th of therapy prior to presentation. Several years ago, he had similar abdominal pain that developed after taking amoxicillin/clavulanic acid but did not seek medical attention at that time and the pain resolved within few days while abstaining from food intake.		
Summaries from Initial and Follow-up Visits	Diagnostic Testing	Interventions
Based on clinical presentation and CT findings, patient was diagnosed with mild acute pancreatitis with Bedside Index of Severity in Acute Pancreatitis (BISAP) score of 0 (< 1% risk of mortality), which is characterized by the absence of organ failure and local or systemic complications. During his hospital stay, patient was managed with aggressive IV hydration and pain management with bowel rest of 2 days duration and significant improvement being noticed within 72 h after which patient was discharged home.	Laboratory studies: WBC, amylase, lipase, and CRP.	Discontinuation of offending drug (amoxicillin/clavulanic acid); aggressive IV hydration and pain management with bowel rest of 2 days duration.
No follow-up visits needed	Imaging: Abdominal CT, MRCP, abdominal ultrasound, and endoscopic ultrasonography.	

## Discussion and conclusions

Identifying the cause of acute pancreatitis can be somewhat challenging especially when trying to identify a certain drug as the causative agent. Drugs are responsible for approximately 0.1–2% of acute pancreatitis incidents with most information about drug-induced pancreatitis being collected from case reports and case series which means that true incidence can be even higher [1–4]. There is no one main mechanism behind drug-induced acute pancreatitis, but several potential mechanisms are currently based on theories. Of the proposed mechanisms include: pancreatic duct constriction with localized angioedema and arteriolar thrombosis, cytotoxic and metabolic effects, and hypersensitivity reactions [2]. As well as drugs with side effects of hypertriglyceridemia and chronic hypercalcemia that are considered risk factors for acute pancreatitis [2]. The diagnosis of drug-induced acute pancreatitis requires a diagnosis of acute pancreatitis and ruling out all other etiologies. Etiologies that were ruled out in this case comprise all possible causes of pancreatitis: gallstones, biliary sludge and microlithiasis, alcohol, smoking, hypertriglyceridemia, scorpion venom, post endoscopic retrograde cholangiopancreatography (ERCP), hypercalcemia, steroids intake, malignancy, infection, trauma, vascular disease [1–4]. Based on American College of Gastroenterology guidelines, consideration for genetic testing for hereditary pancreatitis is based on expert opinion and warranted for pancreatic cancer patients with a personal history of at least 2 attacks of acute pancreatitis of unknown etiology, a family history of pancreatitis, or early-age onset chronic pancreatitis [5]; therefore, the decision was made by the primary and gastroenterology teams on the case not to forgo with genetic testing to rule out hereditary pancreatitis. Immunoglobulin G4 level was 24 mg/dL (reference range: 1–100 mg/dL) which ruled out autoimmune pancreatitis. The evidence found to implicate a certain drug to the development of acute pancreatitis is often inadequate especially when the mechanism is unknown. Badalov N. et al. proposed a classification system of drug-induced acute pancreatitis. This system was based on the number of case reports found in the literature, the available rechallenge data, latency period and ability to exclude other causes of acute pancreatitis [6]. After reviewing summary of drug induced acute pancreatitis based on drug class, we found that ampicillin and penicillin are considered class IV (single case report published, but neither a rechallenge nor a consistent latency period documented) [6]. If the pancreatitis resolves after discontinuation of the drug, suspicion for drug-induced pancreatitis increases. A firm diagnosis can be reasonably established with a rechallenge of the offending drug that results in the recurrence of pancreatitis symptoms [1–4]. Rechallenge was not done in this case. Very few cases, less than 5 total cases, were documented in the literature regarding ampicillin, penicillin, and amoxicillin/clavulanic acid induced acute pancreatitis with true mechanism still being unidentified [7–11]. Table 2 shows the comparison between our patient’s case with published data in the literature.

**Table 2:** Comparing the case of our patient with published data

Case	Patient	Findings	Drug	Delay between introduction of the drug and pancreatitis	Re-challenge	Outcome
Chams et al. 2018 (our case)	58-year-old male	Elevated amylase, lipase with CT abdomen showing pancreatitis	Amoxicillin-clavulanic acid	On day 9th of antibiotic treatment	Not performed	Clinical improvement with fluid hydration and cessation of antibiotic
Campo et al. 2015 [7]	42-year-old woman	Elevated lipase with CT abdomen showing pancreatitis	Amoxicillin-clavulanic acid	While on antibiotic treatment; unknown duration	Not performed	Clinical improvement with fluid hydration and cessation of antibiotic
Cerezo Ruiz et al. 2015 [8]	48-year-old female	Elevated lipase with US abdomen showing pancreatitis	Amoxicillin-clavulanic acid	*Data unavailable*	*Data unavailable*	Spontaneous resolution
Sammett et al. 1998 [9]	7-year-old male	Elevated amylase, lipase with US abdomen showing pancreatitis	Penicillin	3 weeks prior to diagnosis was treated with 10 days of oral penicillin	Not performed	Clinical improvement with fluid hydration and food restriction
Galindo et al.; 1995 [10]	25-year-old male	Acute pancreatitis and cholestatic cute hepatitis	Amoxicillin-clavulanic acid	After 4 weeks of an antibiotic treatment	Not performed	Clinical improvement with fluid hydration and food restriction
Hanlien 1987 [11]	73-year-old woman	Elevated serum amylase, lipase and urine amylase	Ampicillin	On day 6th of antibiotic treatment	Re-exposure 2 weeks later for treatment of pneumonia, with repeat elevated enzymes on the 4th day	Clinical improvement after discontinuation of antibiotic treatment

Drug-induced acute pancreatitis remains rare but should not be disregarded when medical practitioners are faced with a patient presenting with acute pancreatitis with no obvious cause. Being familiar with reports of drugs causing acute pancreatitis can be helpful in identifying the causality and association with a certain drug. Despite the fact that DIP can have a benign course with good prognosis, fatal outcomes still occur and thus DIP should not be overlooked. This case describes a patient with DIP after the intake of amoxicillin/clavulanic acid and when all other common causes of acute pancreatitis were excluded. We again stress on the importance of identifying and reporting cases of DIP to raise awareness among physicians and clinicians. We also stress on the importance of encouraging scientists and researchers to better understand the mechanism of developing drug-induced acute pancreatitis.

## Abbreviations

BISAP: Bedside Index of Severity in Acute Pancreatitis
Ca: Calcium
CRP: C-reactive protein
CT: Computed tomography
DIP: Drug-induced pancreatitis
ERCP: Endoscopic retrograde cholangiopancreatography
IV: Intravenous
MRCP: Magnetic resonance cholangiopancreatography
TG: Triglyceride
WBC: White blood count
